# Improving physical health assessment of old age inpatients on the Oaks Acute Admission Ward

**DOI:** 10.1192/bjo.2021.536

**Published:** 2021-06-18

**Authors:** Dr Karen Aus, Marilia Gougoulaki, Maja Elia, Emily Wall

**Affiliations:** Barnet, Enfield and Haringey Mental Health Trust

## Abstract

**Aims:**

Old age psychiatry patients are subject to increased frailty, comorbid load and medication adverse events than equivalent older age populations without psychiatric illness. Timely physical health assessment and monitoring is therefore an essential part of treatment provision. The Oaks is a 20-bed old age acute admissions ward in Barnet, Enfield and Haringey Mental Health Trust. With this quality improvement project, we aimed to deliver high-quality assessment and treatment of physical health for our patients.

**Method:**

Using NICE guidelines as a blueprint, we devised a list of parameters essential to the management of old age inpatients. This included blood tests (full blood count, urea and electrolytes, liver function, thyroid function, cholesterol, lipids, iron studies, vitamin D, glycated haemoglobin, prolactin), investigations (imaging, ECG, physical examination, cognitive testing) and assessments (body mass index [BMI], functional review, mobility, Rockwood Frailty Score). The implementation goal was to ensure all parameters were acted on within 24 hours of admission (or 48 hours for patients admitted on weekends).

We initially audited these parameters in patients admitted to the Oaks in October and November 2020 (n = 24). We subsequently collated all parameters into an online spreadsheet, which was distributed to ward medical staff. For each new admission, parameters could be marked as pending or complete. The spreadsheet was reviewed in daily ward handover. Following implementation, we collected data on the parameters for patients admitted in December 2020 and January 2021 (n = 16).

**Result:**

Prior to implementation of the spreadsheet, 42.0% of all parameters had been actioned within 24 hours of admission. Following the implementation of the spreadsheet, 86.2% of parameters had been actioned within 24 hours (mean difference 44.2%, 95% CI 13.5% to 64%, p = 0.006).

In detail, there were significant increases in timely actioning of magnesium (increased by 61.7%, p < 0.001), cholesterol (61.7%, p < 0.001), glycated haemoglobin (65.8%, p < 0.001), vitamin D (65.8%. p < 0.001), prolactin (61.7% p < 0.001), lipids (61.7%, p < 0.001), thyroid function (51.7%, p < 0.01), iron studies (80.9%, p < 0.001), imaging (42.5%, p = 0.01), frailty scores (60.0%, p < 0.01), BMI measurement (55.9%, p < 0.001), and functional review (42.5%, p = 0.01).

**Conclusion:**

Implementation of a monitoring spreadsheet with relevant parameters linked to daily ward handover resulted in widespread and significant improvement in the assessment of physical health among old age psychiatry inpatients.

